# Pomegranate juice anthocyanidins induce cell death in human cancer cells by mobilizing intracellular copper ions and producing reactive oxygen species

**DOI:** 10.3389/fonc.2022.998346

**Published:** 2022-09-06

**Authors:** Mohd Farhan, Asim Rizvi, Ferasat Ali, Aamir Ahmad, Mohammad Aatif, Arshi Malik, Mir Waqas Alam, Ghazala Muteeb, Saheem Ahmad, Awal Noor, Farhan Asif Siddiqui

**Affiliations:** ^1^ Department of Basic Sciences, Preparatory Year Deanship, King Faisal University, Al-Ahsa, Saudi Arabia; ^2^ Department of Kulliyat, Faculty of Unani Medicine, Aligarh Muslim University, Aligarh, India; ^3^ Interim Translational Research Institute, Academic Health System, Hamad Medical Corporation, Doha, Qatar; ^4^ Department of Public Health, College of Applied Medical Sciences, King Faisal University, Al-Ahsa, Saudi Arabia; ^5^ Department of Clinical Biochemistry, College of Medicine, King Khalid University, Abha, Saudi Arabia; ^6^ Department of Physics, College of Science, King Faisal University, Al-Ahsa, Saudi Arabia; ^7^ Department of Nursing, College of Applied Medical Sciences, King Faisal University, Al-Ahsa, Saudi Arabia; ^8^ Department of Medical Laboratory Sciences, College of Applied Medical Sciences, University of Hail, Hail, Saudi Arabia; ^9^ Department of Laboratory and Blood Bank, King Fahad Hospital, Al Ahsa, Saudi Arabia

**Keywords:** anthocyanidin, copper, reactive oxygen species, prooxidant, DNA damage, cell death

## Abstract

Anthocyanidins are the most abundant polyphenols in pomegranate juice. This class of molecules includes Delphinidin (Del), Cyanidin (Cya), and Pelargonidin (Pel). Using prostate, breast and pancreatic cancer cell lines PC3, MDA-MB-231, BxPC-3 and MiaPaCa-2, we show that anthocyanidins inhibit cell proliferation (measured by MTT assay) and induce apoptosis like cell death (measured by DNA/Histone ELISA). Copper chelator neocuproine and reactive oxygen species scavengers (thiourea for hydroxyl radical and superoxide dismutase for superoxide anion) significantly inhibit this reaction thus demonstrating that intracellular copper reacts with anthocyanidins in cancer cells to cause DNA damage *via* ROS generation. We further show that copper-supplemented media sensitizes normal breast epithelial cells (MCF-10A) to Del-mediated growth inhibition as determined by decreased cell proliferation. Copper supplementation results in increased expression of copper transporters Ctr1 and ATP7A in MCF-10A cells, which is attenuated by the addition of Del in the medium. We propose that the copper mediated, ROS-induced mechanism of selective cell death of cancer cells may in part explain the anticancer effects of anthocyanidins.

## Introduction

Ancient systems of medicine, such as Greeko Arab Medicine (Unani Medicine) often advocate food products as medicine. Food, in these systems of medicine, exerts its therapeutic capability by its physiological and bio-molecular activity, amongst other factors. Thus the concept of dietary constituents as therapeutic agents is not new. According to epidemiological research, the consumption of fruits and vegetables is connected with a lower risk of cardiovascular illnesses and certain types of malignancies (1–4). Numerous fruits, vegetables, and nuts are abundant in polyphenolic substances such as flavonoids, tannins, curcuminoids, gallocatechins, stilbenes, and anthocyanidins, among others. The processes behind the wide variety of pharmacological characteristics possessed by plant polyphenols have generated considerable interest. They are identified as naturally occurring antioxidants and have been associated with anticancer properties (4). Recent studies have shown that plant polyphenolics such as curcumin (from turmeric), resveratrol (from red grapes and red wine), epigallocatechin-3-gallate (EGCG) (from green tea), and delphinidin (from pomegranate juice) induce apoptosis in numerous cancer cell lines (5–9). The finding that a number of these polyphenols, including EGCG, gallic acid, and resveratrol, elicit apoptotic cell death in diverse cell lines but not in normal cells is of particular relevance (6–8). Flavonoids (10), tannic acid and its structural constituent gallic acid (11), curcumin (12), gallocatechins (13), and resveratrol (14) have been shown to cause oxidative strand breakage in DNA either alone or in the presence of transition metal ions.

Copper is an essential metal ion present in chromatin and is intimately connected with DNA bases, particularly guanine (15). It is one of the most redox-active metal ions found in living cells. Copper elevation has been observed in a wide variety of malignancies (16), both in humans and in laboratory animals. Interestingly, for human malignancies the elevation of copper has been seen both in the serum and in the tissue (16). The elevation of copper in the malignancy does not depend on the tissue type, but is rather a metabolic characteristic of the malignancy itself (17). Whether this systemic elevation of copper is a cause or an effect of the malignant transformation remains open to debate and investigation.

Most of the pharmacological activities of plant polyphenols are believed to be derived from their capacity to scavenge endogenously generated oxygen radicals or those created by xenobiotics, radiation, etc. However, the antioxidant properties of polyphenolic compounds may not fully explain their chemopreventive benefits (18, 19). The majority of plant polyphenols exhibit both antioxidant and prooxidant characteristics (7, 10), and we have previously hypothesized that the prooxidant action of polyphenolics may be a significant mechanism behind their anticancer and apoptosis-inducing properties (19, 20). This mechanism for the lethal effect of these chemicals on cancer cells would include the mobilization of endogenous copper ions and the resulting prooxidant action.

**Figure 1:**
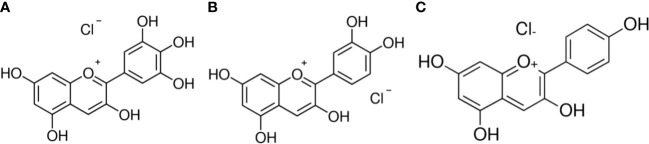
Chemical structures **(A)** Delphinidin **(B)** Cyanidin **(C)** Pelargonidin.

In the present study, we demonstrate that anthocyanidins possess a significant oxidative damage-inducing capacity. We show that these characteristics of anthocyanidins in cancer cells are dependent upon the bioavailability of copper within the cell and its redox recycling. **Figure 1** depicts the structures of the anthocyanidins used in these investigations.

## Material and methods

The immortalized non-transformed breast cell line MCF-10A and cancer cell lines PC3, MDA-MB-231, BxPC-3, and MiaPaCa-2 were obtained from ATCC (Manassas, VA, USA). MDA-MB-231, BxPC-3, and MiaPaCa-2 cell lines were grown in DMEM (Invitrogen, Carlsbad, CA, USA), whilst PC3 cells were grown in RPMI (Invitrogen, Carlsbad, CA, USA). The medium was supplemented with 10% fetal bovine serum (FBS), 100 units/mL penicillin, and 100 g/mL streptomycin. All cells were cultivated, at 37°C and 5% CO_2_. Small aliquots of delphinidin, cyanidin, and pelargonidin stock solutions (50 mM) were kept at -80°C. Neocuproine (Neo), desferoxamine mesylate (DM), and histidine (His) stock solutions were prepared in PBS at a final concentration of 50 mM. MCF-10A, a normal breast epithelial cell line, was grown in DMEM/F12 (Invitrogen, Carlsbad, CA, USA) supplemented with 5% horse serum, 20 ng/mL EGF, 0.5 µg/mL hydrocortisone, 0.1 µg/mL cholera toxin, 10 µg/mL insulin, 100 units/mL penicillin, and 100 µg/mL streptomycin MCF-10A + Cu cells are MCF-10A cells that were grown for one month with 25 µM CuCl_2_ added to their standard culture medium (described above).

### Cell growth inhibition studies by 3-(4,5-Dimethylthiazol-2-yl)-2,5 diphenyltetra-zolium (MTT) assay

In 96-well microtiter culture plates, 2x10^3^ cells per well were seeded. After an overnight incubation, the regular growth medium was replaced with a fresh medium containing various amounts of anthocyanidins diluted from a 50 mM stock. As specified in each study, various chelators were introduced to specific assays. After 3 days of incubation, 25 µl of 3-(4,5-Dimethylthiazol-2-yl)-2,5 diphenyltetrazolium bromide (MTT) solution (5 mg/mL in PBS) was added to each well, and the plates were incubated at 37°C and 5% CO_2_, for an additional 2 hours.

After 2 hours of incubation, the supernatant was discarded. MTT formazan from metabolically viable cells was dissolved in 100 µl of DMSO using a gyratory shaker for 30 minutes. The absorbance was calculated to be 595 nm using an Ultra Multifunctional Microplate Reader (TECAN, Durham, NC, USA). There were eight replicate wells for every treatment, and the concentration of DMSO in the response mixture never exceeded 0.1%. Each experiment was repeated three times.

### Detection of apoptosis through the histone/DNA ELISA

The Cell Death Detection ELISA Kit (Roche, Palo Alto, CA, USA) was used to detect apoptosis in growth cells treated with different anthocyanidins. Cells were treated for 72 hours with polyphenols or DMSO as a control. After treatment, cytoplasmic histone and DNA from cells was extracted and incubated in microtiter plate modules coated with anti-histone antibody. Peroxidase-conjugated anti-DNA antibody was used to detect immobilized histone/DNA, followed by color development with peroxidase-specific ABTS substrate. Using Ultra Multifunctional Microplate Peruser (TECAN, Durham, NC, USA) at 405 nm, the spectrophotometric absorbance of the samples was measured.

In addition, specific metal ion chelators were employed during the processes. DM (50 µM) was employed for the chelation of Fe (II) particles, His (50 µM) was employed for Zn (II), and Neo (50 µM) was employed for the chelation of Cu (II) ions. Catalase 20 µg/mL, superoxide dismutase (SOD) 20 µg/mL, and thiourea (TU) 0.1 mM were used to study the involvement of reactive oxygen species in the reaction.

### Cell migration assay

A cell migration assay was conducted using 24-well transwell permeable supports with 8 mm pores (Corning, NY, USA). The transwell embeds were planted with cells suspended in a solution devoid of serum. The lower wells were filled with media. After 24 hours, cells were stained with 4 mg/mL calcein AM (Invitrogen, Carlsbad, CA, USA) in PBS at 37 C for 1 hour and then detached from the inserts using trypsin. The fluorescence of the migrating cells was measured utilizing the ULTRA Multifunctional Microplate Peruser (TECAN, Durham, NC, USA). Cells were cultured in the presence and absence of delphinidin (50 µM) and neocuprione (50 µM) at various concentrations.

### Real-time reverse transcriptase PCR

Total RNA was isolated using TRIzol reagent (Invitrogen) according to the manufacturer’s instructions. Real-time PCR was used to quantify mRNA expression. Sequences of primers for Ctr1 (forward: 5′-GCT GGA AGA AGG CAG TGG TA-3′; reverse: 5′-AAA GAG GAG CAA GAA GGG ATG-3′), ATP7A (forward: 5′-ACG AAT GAG CCG TTG GTA GTA-3′; reverse: 5′-CCT CCT TGT CTT GAA CTG GTG-3′) and GAPDH (glyceraldehyde-3-phosphate dehydrogenase) (forward: 5′-TGG GTG TGA ACC ATG AGA AGT-3′; reverse: 5′-TGA GTC CTT CCA CGA TAC CAA-3′) were same as reported earlier (21, 22), and the amount of RNA was normalized to GAPDH expression.

### Small interfering RNA (siRNA) transfection

siRNA transfections were carried out as previously described (22). Santa Cruz Biotechnology, Inc. was contacted for Ctr1-specific siRNA. A garbled siRNA was utilized as a control.

Following the manufacturer’s instructions, transfections were carried out with Lipofectamine RNA iMAX Transfection Reagent (Invitrogen), 48 hours before to the experiment, Ctr1 was silenced by siRNA.

### Statistical analysis

The results of all experiments are expressed as percentage of control ± Standard Error (S.E.) of triplicate determinations. p ≤ 0.01 is considered statistically significant.

## Results

### Anthocyanidins suppress cancer cell development and induce apoptosis in several cancer cell types

**Figure 2:**
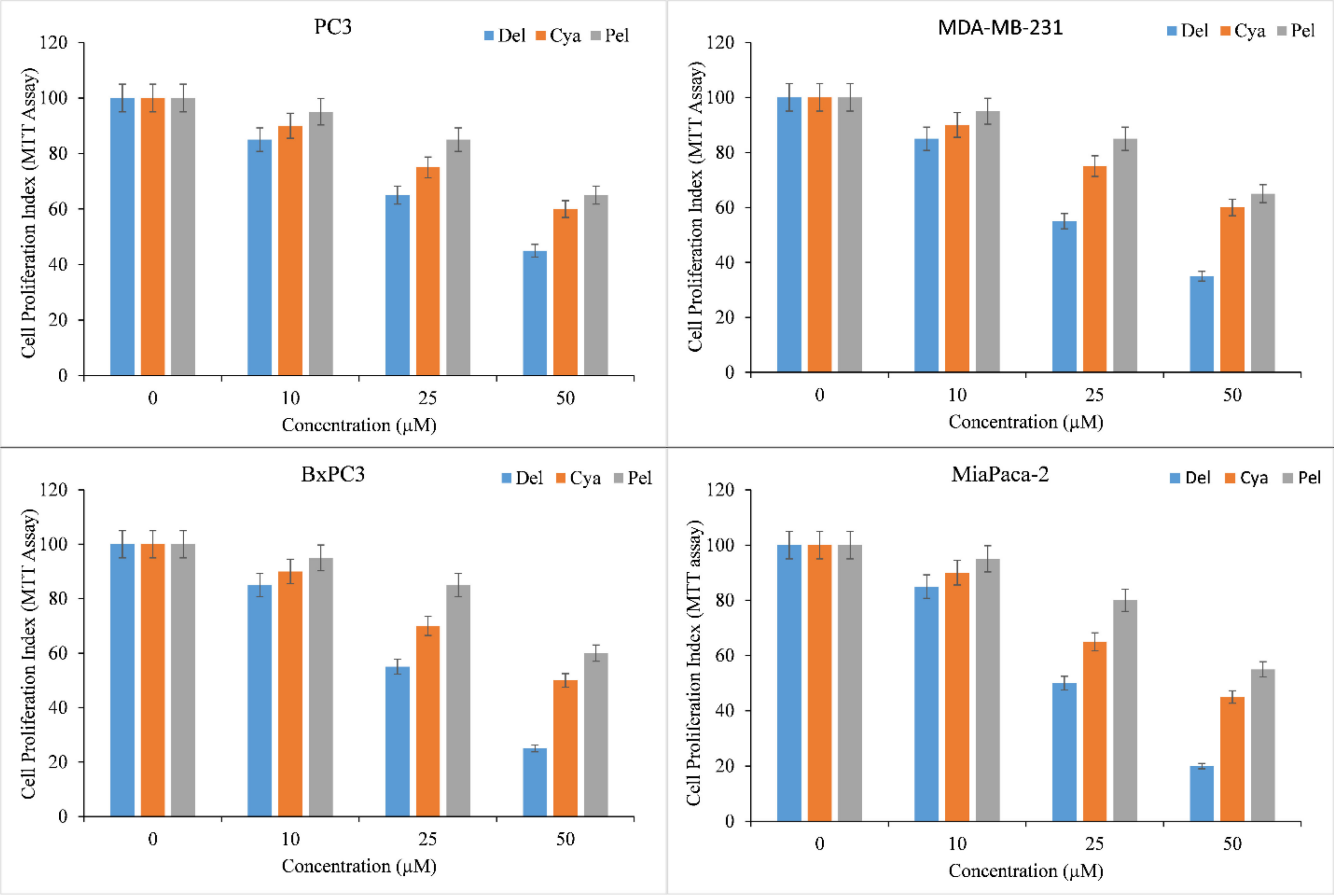
Effect of delphinidin, cyanidin and pelargonidin on cell growth in various cancer cell lines as revealed by MTT assay. Cells from PC3, MDA-MB-231, BxPC3, MiaPaCa-2 cancer cell lines were treated with stated concentrations of anthocyanidins (mentioned above) for 72 hours. The effect on cell proliferation was measured by performing MTT test as indicated in ‘Methods’. All data are expressed as percentage of control ± S.E of triplicate determinations. p ≤ 0.01 when compared to respective untreated control was treated as significant.

Diverse cancer cell lines, PC3 (Prostate), MDA-MB-231 (Breast), and BxPC3 and MiaPaCa-2 (Pancreas), were treated with a range of concentrations of delphinidin, cyanidin, pelargonidin. MTT assay was used to assess the inhibition of cancer cell proliferation by anthocyanidins. All anthocyanidins inhibited cancer cell growth in a concentration-dependent manner (**Figure 2**). However, the inhibition was shown to be significantly stronger in delphinidin than in cyanidin and pelargonidin.

**Figure 3:**
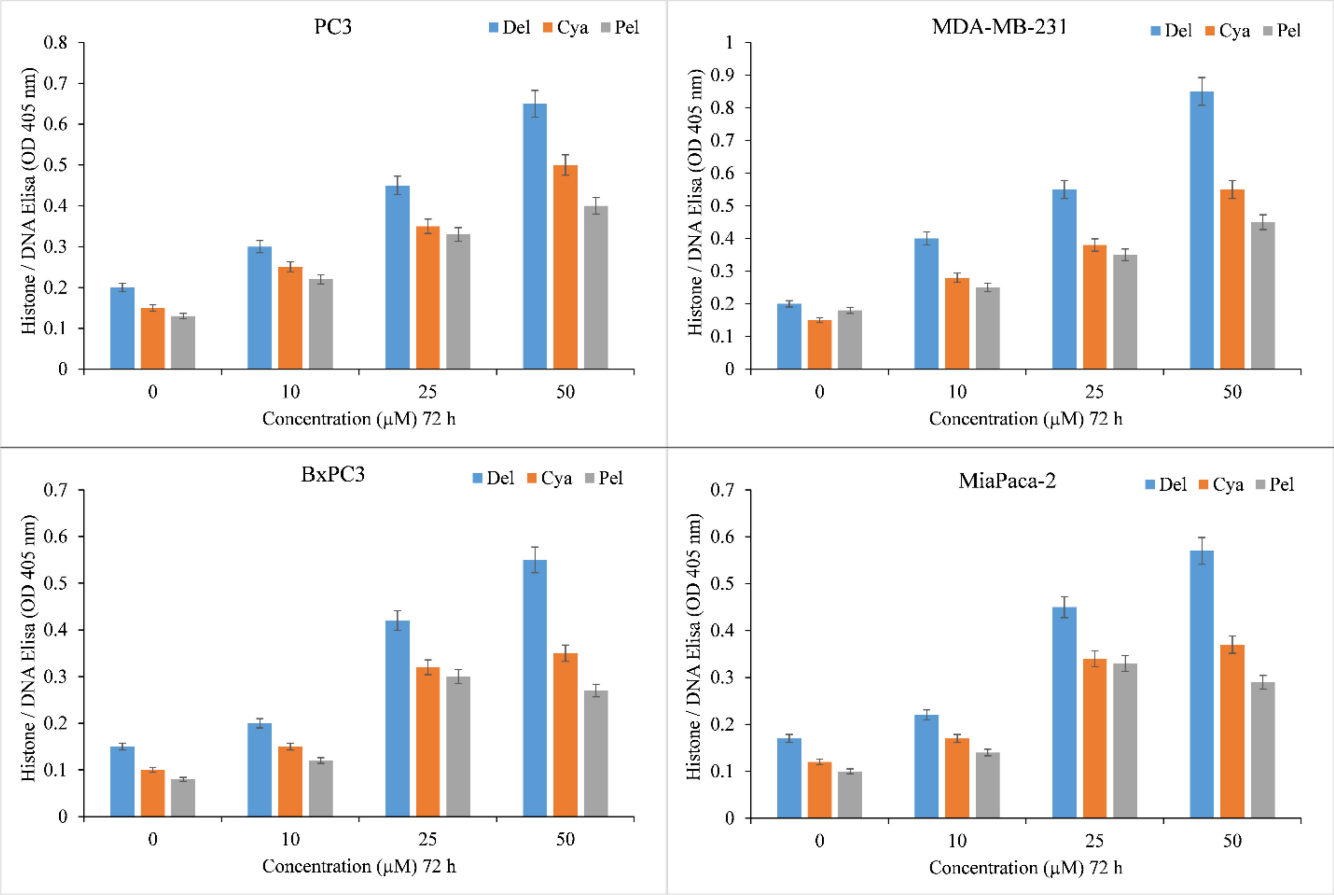
Induction of apoptosis by delphinidin, cyanidin and pelargonidin in several cancer cell lines. The Cell Death Detection ELISA Kit (Roche, Palo Alto, CA) was used to detect apoptosis in cancer cell lines after 72 hours of incubation with increasing concentrations of anthocyanidins (mentioned above), as depicted in the figure and detailed in the section titled “Methods.” The stated values are the S.E. of three separate tests. p ≤ 0.01 when compared to respective untreated control was treated as significant.

The induction of apoptosis by delphinidin, cyanidin, pelargonidin was evaluated by Histone/DNA ELISA to confirm these findings (**Figure 3**). Delphinidin was identified as the most potent molecule, confirming our previous findings. The most efficient inducer of apoptosis was also delphinidin followed by cyanidin and pelargonidin. We thus concluded that the cytotoxicity of anthocyanidins is dose-dependent. Delphinidin was the most effective molecule in this family. Thus for our mechanistic studies we only used Delphinidin.

### Copper chelation decreases the growth inhibition and apoptosis produced by delphinidin

We have previously established that the membrane permeable copper chelator neocuproine inhibits the delphinidin-induced oxidative breaking of cellular DNA in lymphocytes (23), indicating the involvement of endogenous copper in the process. Since lymphocytes differ from cancer cells, we wished to reproduce this effect in malignant cell lines.

**Figure 4:**
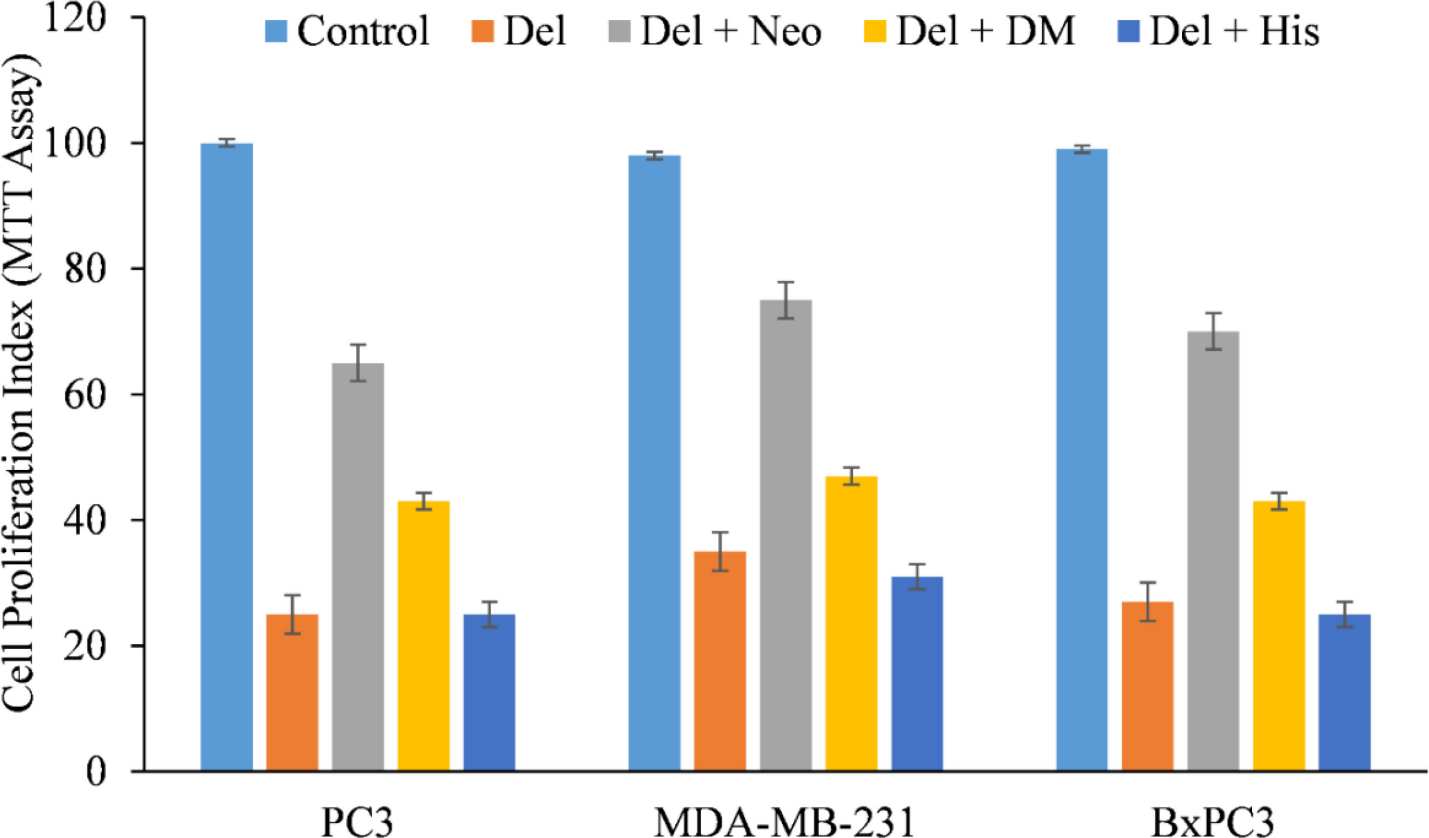
The effect of several metal-specific chelators on the antiproliferative activity of delphinidin in three cancer cell lines is depicted. As depicted in the picture, PC3, MDA-MB-231, and BxPC3 cancer cells were treated with 50 µM of delphinidin alone or in the addition of copper chelator neocuproine (Neo), iron chelator desferrioxamine mesylate (DM), or zinc chelator histidine (His). 50 µM was the concentration of metal chelators utilized. After 72 hours of treatment, the MTT assay was performed as indicated in “Methods.” The values reported are the S.E. of three separate trials. p ≤ 0.01 when compared to respective untreated control was treated as significant.

We therefore, reproduced the investigation with malignant cells and discovered that only copper chelator Neo could significantly protect PC3, MDA-MB-231, and BxPC-3 cells from the growth-inhibiting effects of delphinidin (**Figure 4**). However, DM and His (iron and zinc chelators, respectively) failed to affect cell growth to any substantial degree, with the exception of PC3 and BxPC3 cells, in which DM and His show a protective effect against the growth suppression induced by delphinidin. However, this was significantly less than Neo’s inhibition (**Figure 4**).

**Figure 5:**
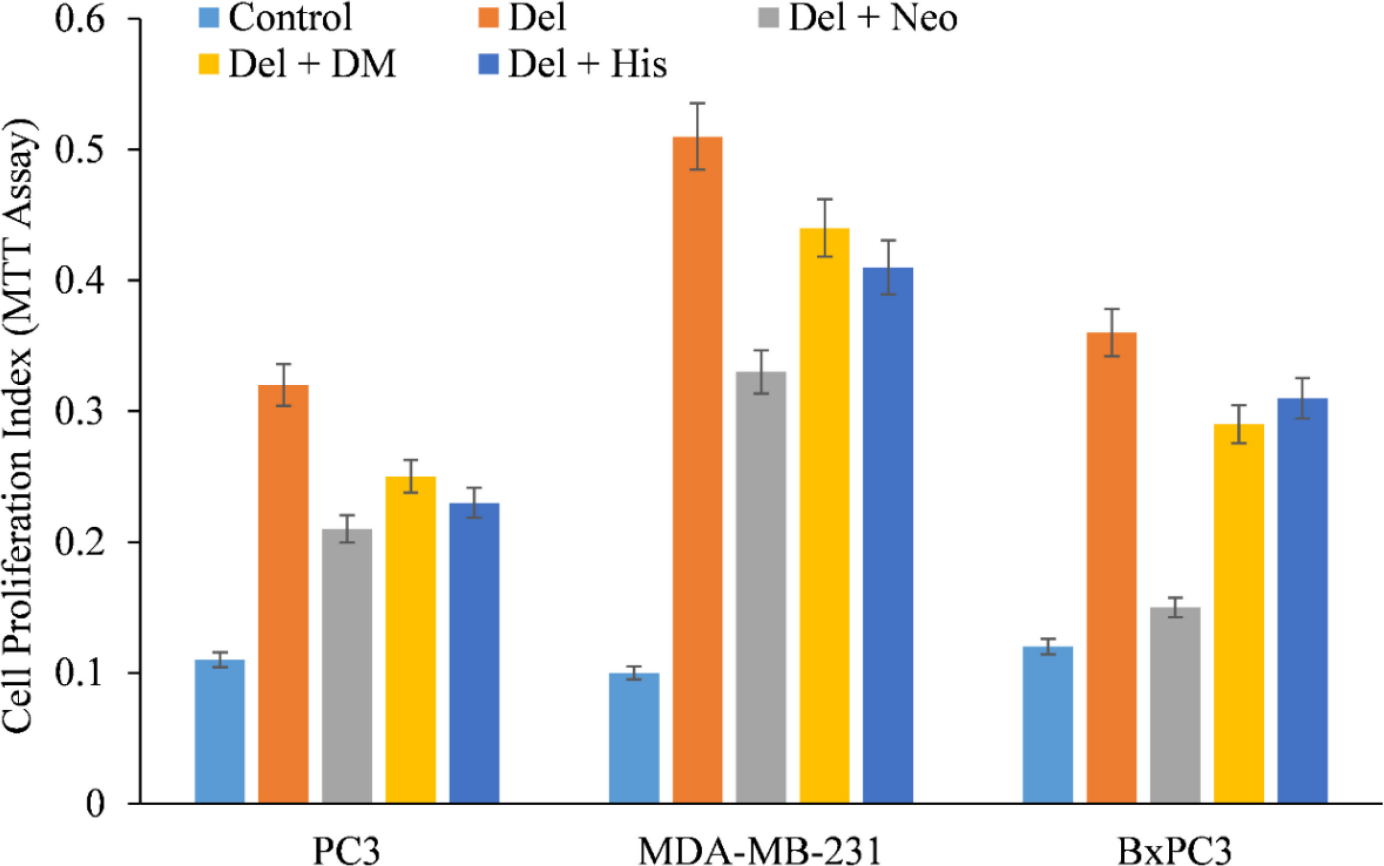
Effect of various metal chelators on delphinidin-induced apoptosis in three distinct cancer cell lines. As depicted in the figure, PC3, MDA-MB-231, and BxPC3 cancer cells were treated with 50 µM of delphinidin alone or in the presence of the copper chelator neocuproine (Neo), the iron chelator desferrioxamine mesylate (DM), or the zinc chelator histidine (His). The concentration of metal chelators utilized was 50 µM. After 72 hours of treatment, MTT assay was performed as indicated in “Methods.” p ≤ 0.01 when compared to respective untreated control was treated as significant.

Additionally, the impact of several metal chelators on delphinidin-induced apoptosis was evaluated. This protection was not detected when iron or zinc chelators were used (**Figure 5**), corroborating the conclusion that the anticancer mechanism of the drug delphinidin includes mobilization of endogenous copper.

Polyphenol induced apoptosis of cancer cells is caused by ROS mediated DNA breakage (24, 25). To determine whether anthocyanidin-induced DNA damage in cancer cell lines also involved ROS, the effect of several ROS scavengers (such as catalase, thiourea, and superoxide dismutase) on delphinidin-induced death of cancer cells was also investigated. All three ROS scavengers inhibited delphinidin-induced apoptosis in diverse cancer cell lines (**Table 1**), with TU exhibiting the greatest degree of inhibition. These results validated the significance of reactive oxygen species (ROS) as effectors of anthocyanidin-induced apoptosis, perhaps by a Fenton-type physiologically active reaction, as described previously (26, 27).

**Table 1:** The effect of ROS scavengers on the activation of apoptosis in two distinct cancer cell lines by delphinidin.

Cancer Cell Line	Dose	Apoptosis (folds)	Effect of Scavengers
**PC3**	Untreated	–	–
	Delphinidin (50 µM)	2.49	–
	Thiourea	1.78	28.51
	Catalase	2.13	14.45
	SOD	1.97	20.88
**MDA-MB-231**	Untreated	–	–
	Delphinidin (50 µM)	3.32	–
	Thiourea	2.17	34.63
	Catalase	2.69	18.97
	SOD	2.51	24.39

Along with delphinidin, cancer cells were co-incubated with multiple ROS scavengers, including TU, (100 µM Thiourea); Cat, (100 mg/mL catalase); and SOD, (100 mg/mL superoxide dismutase); The effect on apoptosis was determined using Histone/DNA ELISA as described in the “Methods” section. Apoptosis (folds) represents the increase in apoptosis in comparison to the untreated control.

### Copper chelation destroys delphinidin inhibited the migration of cancerous cells

**Figure 6:**
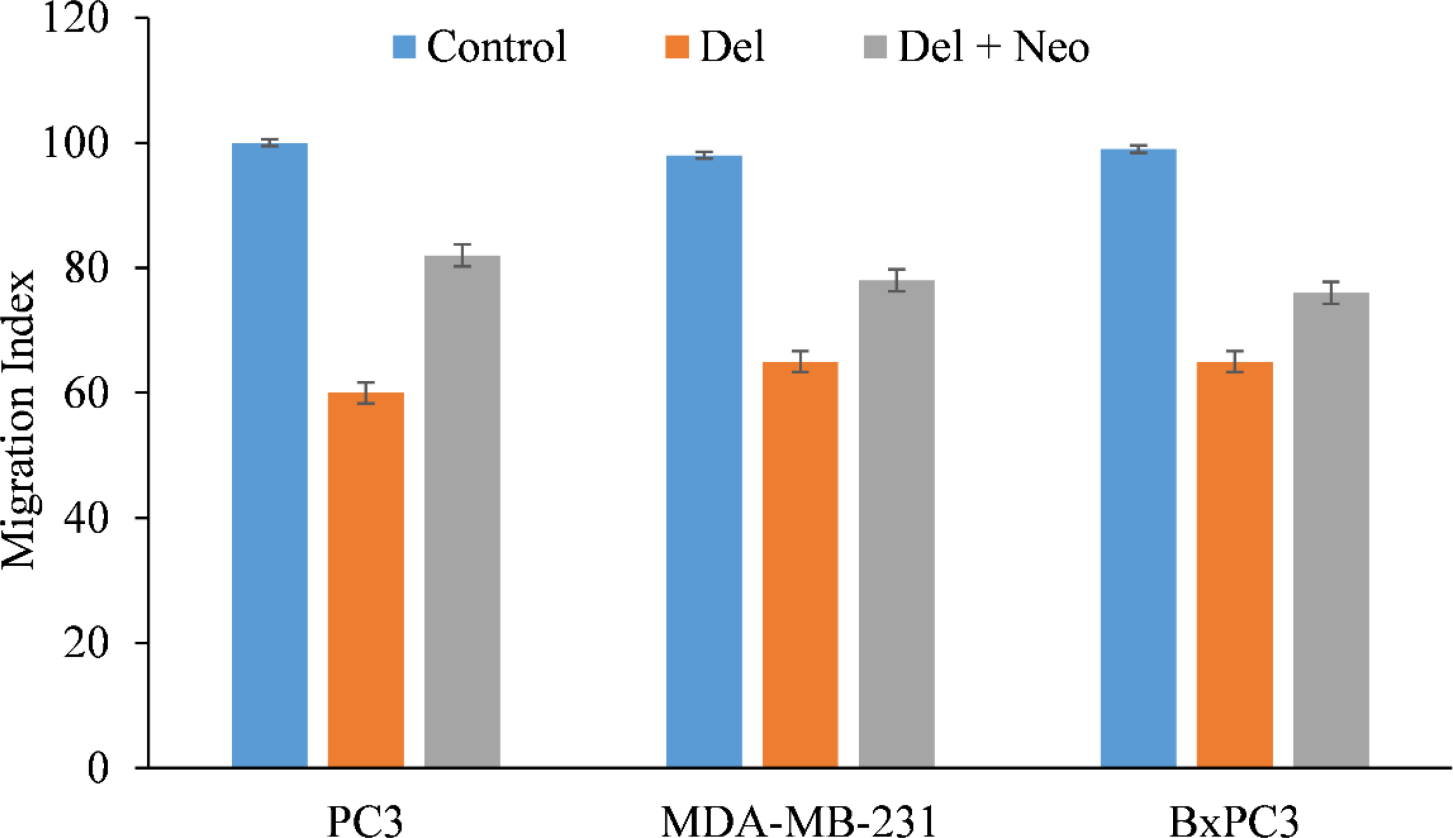
In the presence of the copper chelating agent neocuproine, the effect of delphinidin on the migration of PC3 (prostate), MDA-MB-231 (breast), and BxPC3 (pancreatic) cancer cells was examined. As detailed in “Methods,” a cell migration test was conducted utilizing 24-well transwell permeable supports with 8-mm pores (Corning). In the presence and absence of delphinidin (50 µM) and neocuprione (50 µM), the cells were developed. Ultra Multifunctional Microplate Reader was used to analyze the fluorescence of the migrating cells (TECAN). The values reported are the S.E. of three separate trials. p ≤ 0.01 when compared to respective untreated control was treated as significant.

Malignant cells are characterized by their migration and invasion of secondary locations *via* metastasis. We found that delphinidin decreased the migratory capacity of PC3 and MDA-MB-231, cells (**Figure 6**), making them less susceptible to metastasis. Interestingly, when copper was chelated from the cells by the membrane permeable copper chelator neocuprione in the presence of delphinidin, the cells regained their metastatic potential (**Figure 6**).

### Copper supplementation makes normal breast epithelial cells more sensitive to the antiproliferative effects of delphinidin

**Figure 7:**
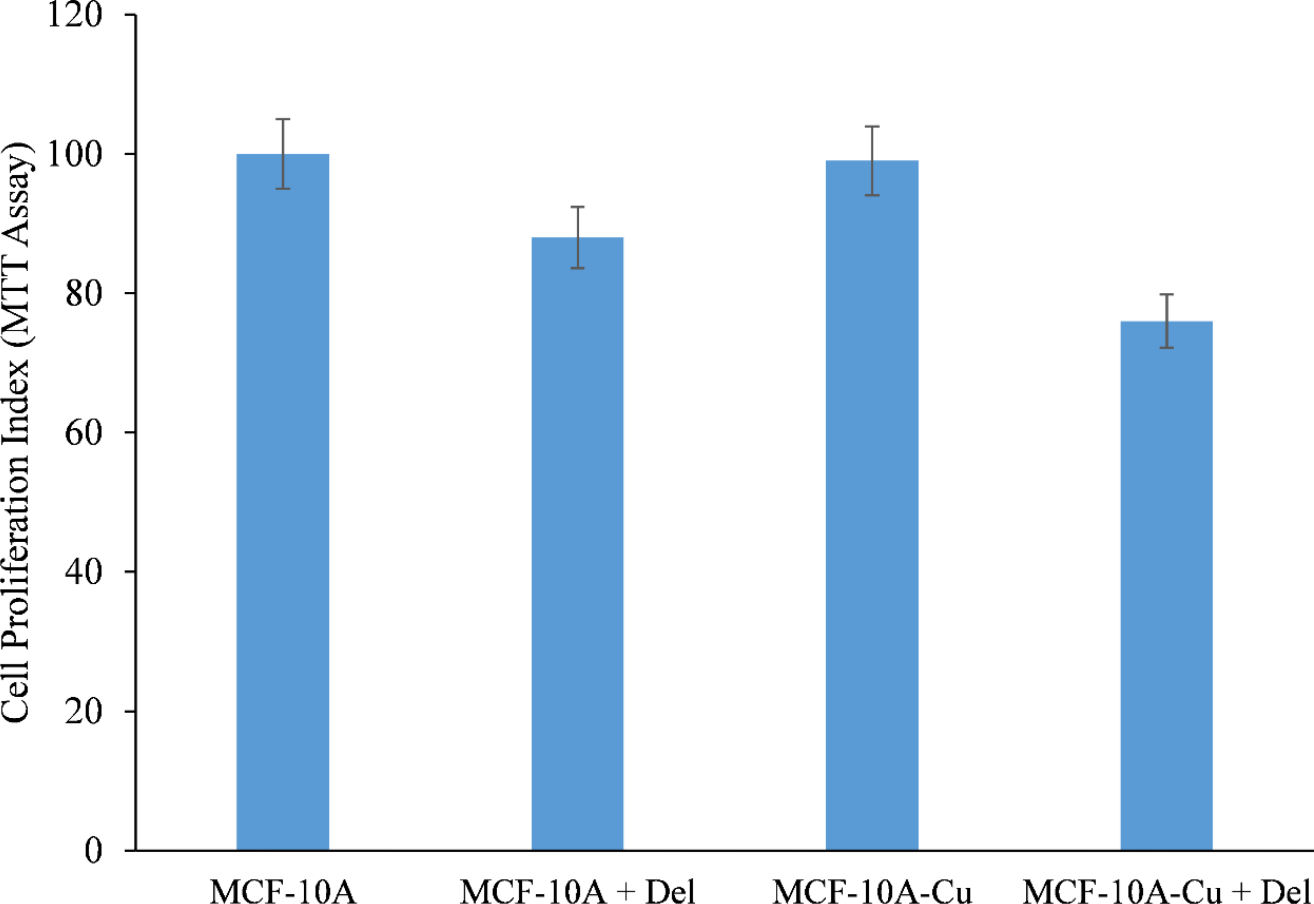
Effect of delphinidin on cell growth suppression in MCF-10A (normal breast epithelial cells) and MCF-10A cells cultivated in Cu (II)-supplemented medium (MCF-10A-Cu). MCF-10A and MCF-10A-Cu (normal cells cultivated in a medium containing 25 µM CuCl_2_) were treated with delphinidin 50 µM for 72 hours. The proliferation of cells was then measured using the MTT test, as stated in “Methods.” The stated values are the S.E. of three separate experiments. p ≤ 0.01 when compared to respective untreated control was treated as significant.

MCF-10A normal (non-cancerous) breast epithelial cells were grown in medium containing 25 µM copper. When copper-supplemented MCF-10A cells (MCF-10A+Cu) were treated with delphinidin, a substantial decrease in cell proliferation was found as compared to non-copper-supplemented MCF-10A cells (**Figure 7**).

Since malignant transformation is followed by a dramatic increase in intracellular copper levels (24, 26, 27), it is plausible to conclude that the delphinidin-induced inhibition of malignant cell proliferation is a result of its interaction with cellular copper. The addition of exogenous copper to non-malignant epithelial cells sensitizes these non-malignant cells to delphinidin-induced cell growth suppression (**Figure 7**).

### Delphinidin inhibits copper transporter Ctr1 and ATP7A expression

**Figure 8:**
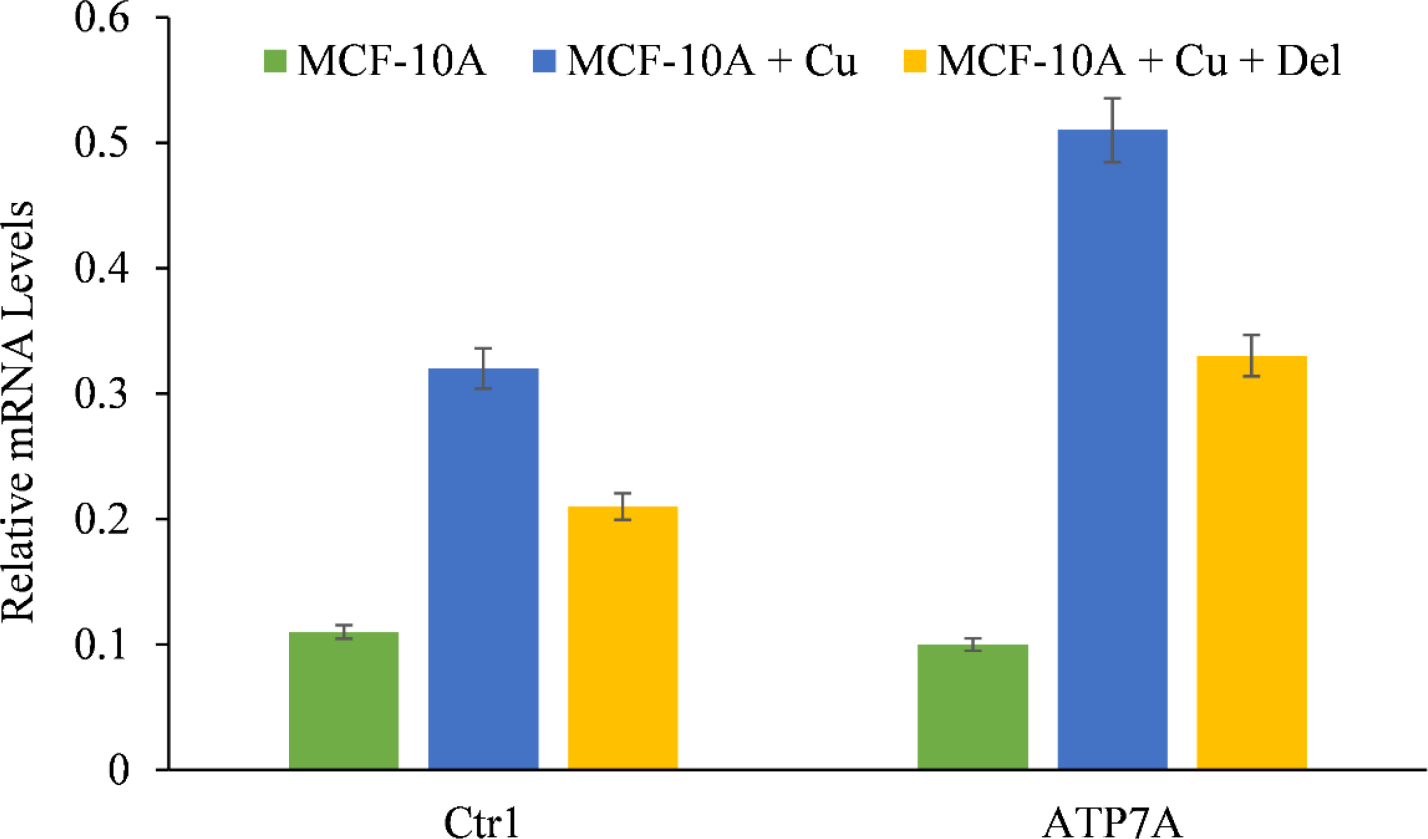
The effect of delphinidin on the elevated mRNA transcript levels of copper transporters Ctr1 and ATP7A in MCF-10A-Cu cells compared to their parental MCF-10A cells. TRIzol (Invitrogen) was used to isolate total RNA in accordance with the manufacturer’s instructions. As described in “Methods,” Ctr1 and ATP7A mRNA expression was quantified using real-time PCR. The values reported are the S.E. of three separate trials. p ≤ 0.01 when compared to respective untreated control was treated as significant.

We showed that the growth inhibition elicited by delphinidin is a result of its interaction with intracellular copper in both malignant cells (**Figures 4**, **5**) and non-malignant epithelial cells cultured in media enriched with copper (**Figure 7**). Since malignant cells express copper transporter Ctr1 at a higher level (28), we next examined whether copper supplementation led to an increase in copper transporter expression in non-malignant epithelial cells. Copper supplementation in the growth media of MFC-10A cells led to a significant increase in the expression of copper transporters Ctr1 and ATP7A, (**Figure 8**) (28, 29). Adding more delphinidin to the medium decreased the expression of both copper transporters (**Figure 8**), indicating that delphinidin had an influence on the copper metabolism of cancer cells.

### Targeted silencing of Ctr1 in MCF-10A cells cultured in copper-supplemented media decreases the suppression of proliferation produced by delphinidin

**Figure 9:**
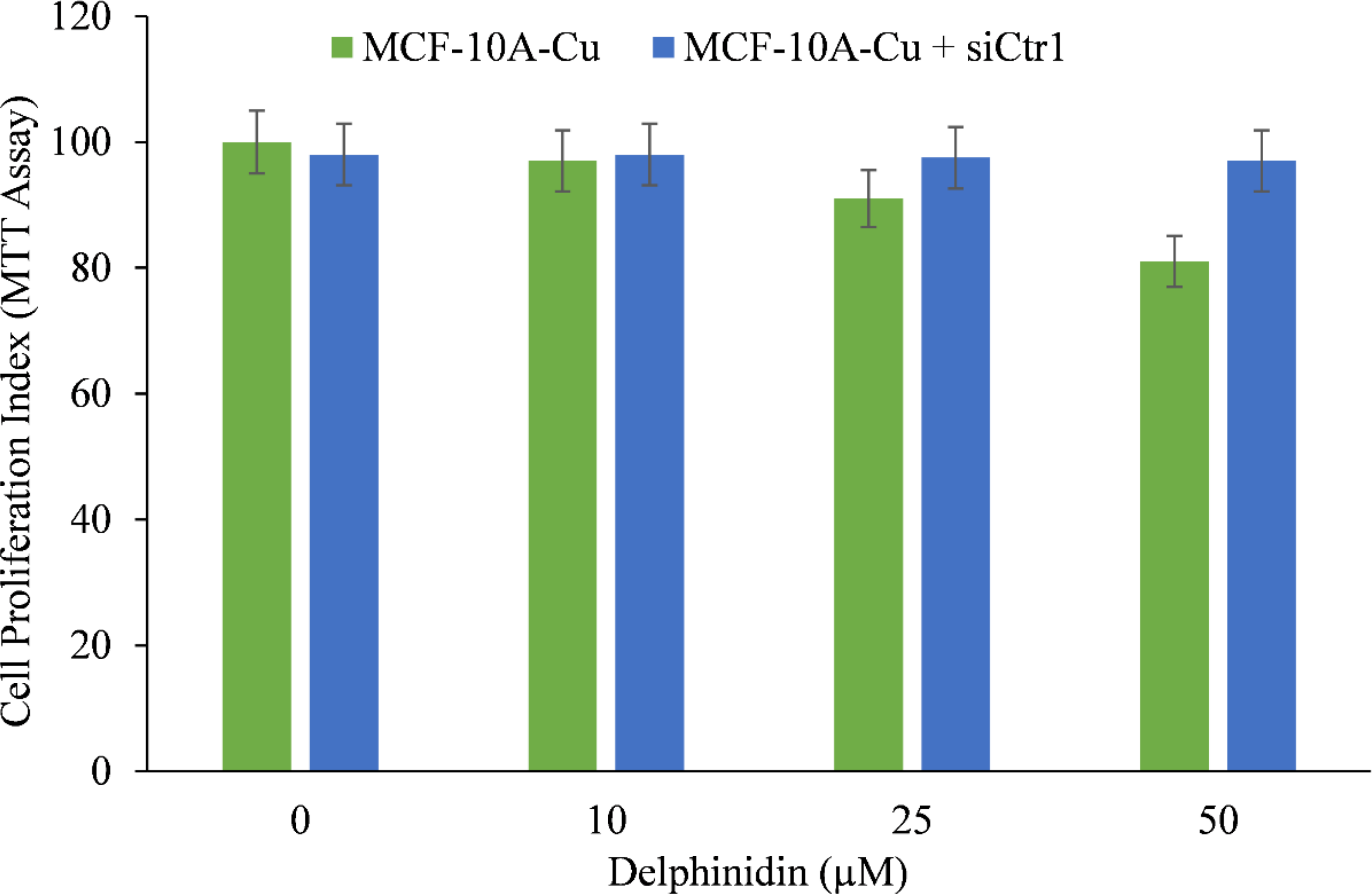
The effect of delphinidin on the elevated mRNA transcript levels of copper transporters Ctr1 and ATP7A in MCF-10A-Cu cells compared to their parental MCF-10A cells. TRIzol (Invitrogen) was used to isolate total RNA in accordance with the manufacturer’s instructions. As described in “Methods,” Ctr1 and ATP7A mRNA expression was quantified using real-time PCR. To determine the effect of delphinidin on mRNA expression, only MCF-10A-Cu cells (normal MCF-10A cells cultured in a medium containing 25 µM CuCl2) with enhanced mRNA expression of copper transporters were treated with 50 µM delphinidin. The values reported are the S.E. of three separate trials. p ≤ 0.01 when compared to respective untreated control was treated as significant.

We wished to validate the crucial involvement of copper in the growth inhibition mediated by delphinidin. To this end we silenced the copper transporter Ctr1 using targeted siRNA (**Figure 9**). Ctr1 mediates copper uptake in cells, and as established before (**Figure 8**), its expression makes MCF-10A cells more susceptible to delphinidin-induced growth suppression. We discovered that silencing copper transporter Ctr1 decreased MCF-10A cells’ susceptibility to delphinidin when cultured in copper-enriched media.

This demonstrates conclusively that delphinidin interacts with cellular copper and that cellular copper is essential for the growth-inhibiting effect of delphinidin on cancer cells.

## Discussion

Similar to other classes of plant polyphenols, the anthocyanidin (delphinidin, cyanidin and pelargonidin) are capable of causing (i) cellular DNA degradation in human cancer cell lines either alone or in the presence of copper ions (ii) such DNA breakage is caused by the production of reactive oxygen species (ROS) and (iii) delphinidin is most capable of causing DNA breakage (among the anthocyanidins tested) in the cancer cell lines tested.

Pomegranate has been utilized in traditional medicine for millennia, and is now recognized as a possible anticancer agent. In animal models, pomegranate juice has been demonstrated to be cytotoxic against skin, prostate, lung, breast, colon, and blood malignancies. On consumption of pomegranate juice, a variety of proteins and transcription factors implicated in apoptosis induction pathways are up- or down-regulated (30). The principal anthocynanidin in pomegranate juice, delphinidin, has been demonstrated to cause apoptosis in human pro-myelocytic leukemia (HL-60) cells (9). It was demonstrated that a JNK activation pathway driven by oxidation may be implicated in delphinidin-induced apoptosis. In addition, delphinidin caused the cells to create hydrogen peroxide, and antioxidants such as N-acetylcysteine and catalase reduced JNK phosphorylation, caspase-3 activation, and DNA fragmentation induced by delphinidin. According to structure–activity studies, the presence of orthodihydroxy phenolic groups on the B-ring of anthocyanidins is important for inducing apoptosis. It is noteworthy that we have previously demonstrated that such a structural configuration of hydroxyl groups in flavonoids and tannins is crucial for the oxidative destruction of DNA in the presence of copper ions (11, 31–33). Gallic acid (which contains ortho dihydroxy phenolic groups) is also active in lymphocyte DNA destruction, although its modified derivative syringic acid is not. Antioxidants, such as catalase, superoxide dismutase, and hydroxyl radical scavengers, also suppress this type of cellular DNA breaking (34, 35).

The bioavailability of delphinidin in mammalian systems warrants discussion. It has been observed that delphinidin and cyanidin decrease vascular endothelial growth factor (VEGF) expression in vascular smooth muscle cells at doses as low as 10 µM (36). It has not escaped our notice that 10 µM delphinidin (without additional copper) is capable of causing considerable DNA damage in MCF-10A cancer cell line (**Figure 7**). The concentration of cyanidin and delphinidin in red wine is approximately 150 µM (37) and the bioavailability of cyanidin and delphinidin has been estimated to be approximately 60% in healthy volunteers after red wine consumption, and therefore such concentrations are likely to be achieved in the blood after red wine consumption (38). Another future area of research can be to determine if anthocyanidins, used in this study can produce a synergistic chemotherapeutic effect with other naturally derived molecules or chemotherapeutic drugs. Other areas where research on dietary polyphenols and their anticancer effects can be extrapolated has been discussed by us elsewhere in great details (25).

The elevation of copper in malignancies is a well-established fact, which is supported by adequate experimental and clinical evidence. This idea is also validated by complementary and alternative systems of medicine, such as Greeko Arab Medicine (Unani Medicine), where it is postulated that elevation in physiologically available metals can increase the propensity of a tumor mass to be cancerous.

We have previously shown (19, 20) that the prooxidant activity of plant polyphenols involving the mobilization of endogenous copper ions may be a key mechanism underlying their anticancer activities. According to our hypothesis, the preferential cytotoxicity of plant polyphenols towards cancer cells is explained by the observation made decades earlier that serum (39, 40), tissue (41), and intracellular copper levels in cancer cells (42) are significantly elevated in a variety of cancers. Since cancer cells possess elevated levels of copper, they may be more susceptible to electron transfer with polyphenols to produce reactive oxygen species (43). As a result of higher intracellular copper levels in cancer cells, it is possible to expect that the lethal concentrations of polyphenols necessary for these cells would be lower than for normal cells (44, 45).

In animal studies, plant polyphenols have been demonstrated to trigger tumor regression (46–48). Our own *in vivo* studies on hepatocellular carcinoma show that EGCG is capable of inducing cell death of malignant cells (49), a similar mechanism has also been demonstrated by Rizvi et al. using Vitamin D (27).

Thus, plant polyphenols with anticancer and apoptosis-inducing qualities might mobilize endogenous copper ions. Our experiments here demonstrate that thiourea, a chelator of hydroxyl radical, superoxide dismutase, a scavenger of superoxide free radical and catalase, a scavenger of hydrogen peroxide, reduces the ability of anthocyanidins to cause copper mediated cell death, possibly, by reducing the generation of ROS near DNA (50, 51).

This mechanism would thus essentially be an alternate, non-enzymatic copper-dependent mechanism for the cytotoxic activity of some anticancer molecules capable of mobilizing endogenous copper. Indeed, a shared mechanism, common to all polyphenols, such as the mechanism elucidated here, better explains the anticancer effects of polyphenols with varied chemical structures.

In live cells, the most redox-active metal ions are Fe^3+^ and Cu^2+^. Although iron is substantially more prevalent in biological systems, copper and zinc are the predominant ions in the nucleus (52). Burkitt et al. (53) listed various reasons why Cu^2+,^ and not Fe^3+^, may be responsible for OP-stimulated DNA fragmentation in isolated nuclei.

ROS production in normal cells is tightly regulated by homeostasis (54). Numerous variables, including metals, medicines, and prooxidants such as H_2_O_2_, can increase ROS production, leading in oxidative stress and the activation of apoptosis. Several investigations have shown that the activation of apoptosis by polyphenols and other anticancer drugs is independent of caspases and mitochondria (55, 56) and is associated by an increase in the intracellular levels of reactive oxygen species (ROS) (57–59).

Copper transporters enable intracellular copper levels to increase. This plays a critical role in the anticancer activity of anthocyanidins and plant-derived polyphenols in general. This study adds a new dimension to the designing of future mechanism-based anticancer lead molecules, which target the tumor microenvironment, specifically increased cellular copper, in order to achieve the desired efficacy.

## Data availability statement

The original contributions presented in the study are included in the article/Supplementary Material. Further inquiries can be directed to the corresponding author.

## Author contributions

All authors listed have made a substantial, direct, and intellectual contribution to the work, and approved it for publication.

## Funding

This work was supported by the Deanship of Scientific Research, Vice Presidency for Graduate Studies and Scientific Research, King Faisal University, Saudi Arabia (Project Number GRANT 1370).

## Conflict of interest

The authors declare that the research was conducted in the absence of any commercial or financial relationships that could be construed as a potential conflict of interest.

## Publisher’s note

All claims expressed in this article are solely those of the authors and do not necessarily represent those of their affiliated organizations, or those of the publisher, the editors and the reviewers. Any product that may be evaluated in this article, or claim that may be made by its manufacturer, is not guaranteed or endorsed by the publisher.
